# Determination of Fluoxetine in Weight Loss Herbal Medicine Using an Electrochemical Sensor Based on rGO-CuNPs

**DOI:** 10.3390/molecules28176361

**Published:** 2023-08-30

**Authors:** Aline Giuli Melaré, Francisco Contini Barreto, Martin Kassio Leme Silva, Rafael Plana Simões, Ivana Cesarino

**Affiliations:** Department of Bioprocess and Biotechnology, School of Agriculture, São Paulo State University, Botucatu 18610-034, SP, Brazil; aline.melare@unesp.br (A.G.M.); francisco.c.barreto@unesp.br (F.C.B.); martin.leme@unesp.br (M.K.L.S.); rafael.simoes@unesp.br (R.P.S.)

**Keywords:** electrochemical sensors, reduced graphene oxide, copper nanoparticles, herbal medicine, fluoxetine

## Abstract

The rising popularity of herbal medicine as a weight loss remedy, fueled by misleading propaganda, raises concerns about the manufacturing processes and potential inclusion of controlled substances such as fluoxetine (FLU). The objective of this work is to develop and evaluate the performance of an electrochemical device by modifying a glassy carbon electrode (GC) with a nanocomposite based on reduced graphene oxide (rGO) and copper nanoparticles (CuNPs) for detecting FLU in manipulated herbal medicines. Scanning electron microscopy (FEG-SEM) and cyclic voltammetry (CV) were applied for morphological and electrochemical characterization and analysis of the composite’s electrochemical behavior. Under optimized conditions, the proposed sensor successfully detected FLU within the range of 0.6 to 1.6 µmol L^−1^, showing a limit of detection (LOD) of 0.14 µmol L^−1^. To determine the presence of FLU in herbal samples, known amounts of the analytical standard were added to the sample, and the analyses were performed using the standard addition method, yielding recoveries between −2.13 and 2.0%.

## 1. Introduction

By reviewing the behavior of humanity throughout the centuries, it is evident that society possesses fluidity, dynamism, and adaptability in order to respond to historical contexts. An example of this dynamic nature is evident in the fluctuating beauty standards across different eras. In the Middle Ages, larger bodies were considered symbols of power, beauty, and good health. However, modern societies idolize thin bodies while associating fat bodies with unattractiveness and poor health [1]. The media, particularly social media, has played a significant role in promoting these ideals and has become an informal source of health education. Presently, a weight-centric approach to health emphasizes the importance of weight management for overall well-being [2]. Numerous studies indicate that exposure to images of thinner bodies leads to increased dissatisfaction with one’s own body among women [3,4,5,6]. Consequently, many individuals’ resort to extreme methods to rapidly lose weight and conform to societal norms, such as prolonged fasting, laxatives use, and medication intake [7].

In recent years, phytotherapy has gained popularity due to its cost-effectiveness and minimal side effects. However, misusing these medications, often taken without medical supervision, can result in adverse effects and, in extreme cases, even lead to death [7,8]. There has been a rise in advertisements for phytotherapy products promising miraculous weight loss. Despite the availability of these plant-based medications without a prescription, consumers frequently purchase them through informal channels such as beauty salons, gyms, and the Internet [9]. This raises concerns as the origin and authenticity of these medications are unknown. Instances of adulteration and tampering with botanical medicines have become increasingly common [10,11,12,13,14]. Illegally adding synthetic drugs, including stimulants, appetite suppressants, and antidepressants such as fluoxetine, is frequently reported in Brazil and other countries. Moreover, these substances are often not listed on the product labels [15]. Ingesting these controlled substances unknowingly can lead to dangerous drug interactions and compromise the health of individuals [16].

Electrochemical sensors offer a low-cost, fast, and straightforward method of analysis. Their high sensitivity, potential for miniaturization, automation, and ability to utilize modified electrodes that enhance sensitivity and lower detection limits have made them extensively researched and used [17]. Carbon-based nanostructured materials are widely employed for electrode modification, as they improve reactivity, sensitivity, and selectivity [18]. Graphene possesses desirable characteristics with its molecular barrier properties, mechanical strength, and electrical conductivity. However, the challenges associated with its synthesis, solution agglomeration, and poor solubility have hindered its practical use. One approach to overcome these obstacles is the synthesis of graphene-like compounds from graphite. Graphite oxide, obtained by oxidizing graphite in protonated solvents, consists of multiple stacked layers of graphene oxide (GO). GO retains a hexagonal structure similar to graphene but contains more oxygen-based functional groups. Compared to pristine graphene, GO offers advantages such as increased solubility and a potential for surface functionalization. To achieve properties closer to pristine graphene, reduced graphene oxide (rGO) can be synthesized [19].

Additionally, metallic nanoparticles such as gold, silver, copper, and antimony are commonly employed as modifiers in electrochemical sensors due to their high chemical stability, good conductivity, and large surface area [20,21]. Copper nanoparticles, specifically, have some particularities that are of interest for electrochemical sensing applications, such as its electrical and catalytic properties, due to a large surface-to-volume ratio. Furthermore, when integrated with carbonaceous materials, such as rGO, the CuNPs enhance the charge transfer between analytes and support matrices, improving the sensor’s performance [22,23].

Numerous studies on the detection of fluoxetine with electrochemical sensors can be found in the literature. However, most of the times, these studies are conducted using water as a sample. Nevertheless, it is also possible to find studies that conducted its experiments in serum samples, blood serum, spiked plasma, and urine [24,25,26,27,28]. This work differs from the ones found in the literature because herbal medicine is not commonly used as the sample, and, since there have been numerous reports that show the presence of fluoxetine in these plant-based medicines [29,30], it is important to verify how an electrochemical sensor behaves with the herbal medicine being used as the sample, since its matrix can interfere with the electrode due to its complexity.

This study focuses on using rGO modified with copper nanoparticles (rGO-CuNPs) to quantify fluoxetine in herbal drugs. The sensor’s performance was characterized using cyclic and differential pulse voltammetry, yielding satisfactory results. This research contributes to the identification of and the combat against the sale of irregular medications by proposing a new, simple detection method.

## 2. Results and Discussion

### 2.1. Morphological and Electrochemical Characterization of the Materials

**Figure 1:**
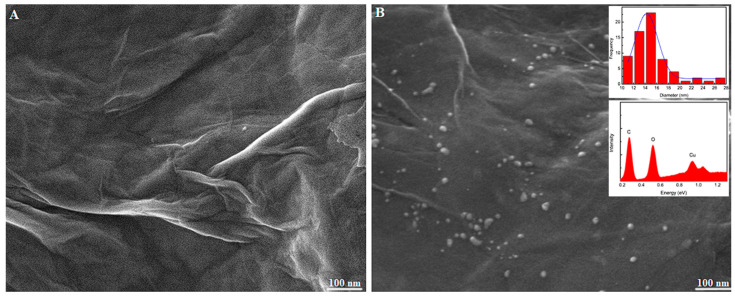
FEG-SEM images of (**A**) rGO and (**B**) rGO-CuNPs (inset: histogram and EDS spectrum of the nano-particle diameters).

The materials’ morphological characterization was carried out using a Field Emission Gun–Scanning Electron Microscope (FEG-SEM). Figure 1A shows the rGO sheet with wrinkled and ripple structures, indicating that, during the GO’s reduction reaction, a modification of its surface occurred, unstacking the GO’s sheets, unblocking its active site, and increasing its surface contact.

**Figure 2:**
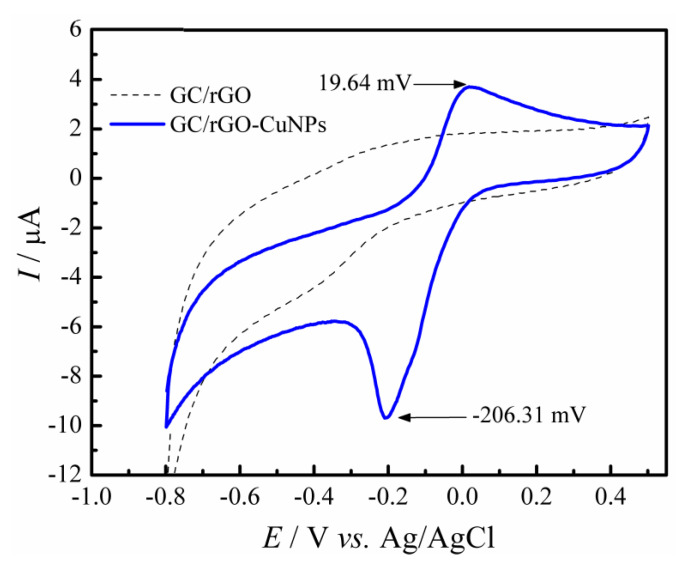
Cyclic voltammograms recorded with GC/rGO (dashed line) and GC/rGO-CuNPs (solid line) in 0.1 mol L^−1^ PBS pH 7.0. *v* = 50 mV s^−1^.

In Figure 1B, on the other hand, it is possible to see the incorporation of copper nano-particles (CuNPs) on the rGO sheet, with diameters between 10 nm and 28 nm. By incorporating metallic nanoparticles into materials such as graphene, there is an increase in the surface area and a subsequent amplification of the electrochemical response due to the synergetic effect between the two nanomaterials that may lead to improved analytical features of the analyte of interest. To confirm the presence of Cu in the composite, EDS was carried out. The inset in Figure 2 shows that the element was incorporated successfully into the material.

Cyclic Voltammetry experiments were carried out for the electrochemical characterization of the GC/rGO-CuNPs, in 0.1 mol L^−1^ PBS (pH 7.0) (*v* = 50 mV s^−1^; potential window = 0.5 V to −0.8 V). When comparing the electrochemical behavior of GC/rGO and GC/rGO-CuNPs, the first one did not show any voltammetric response, as represented by the dashed line in Figure 2. In contrast, for the GC/rGO-CuNPs electrode, its voltammetric profile exhibited the electrochemical processes regarding the copper nanoparticles, with an oxidation peak of the Cu ions defined at 19.64 mV and the reduction process at—206.31 mV, which was attributed to the reduction of the Cu^2+^ to Cu^0^. Considering that the electrochemical process observed on the electrode surface is typical of these nanoparticles [31,32,33,34,35,36], it can be inferred that the CuNPs were successfully incorporated on the GC/rGO.

### 2.2. Electrochemical Behavior of the Modified Electrodes

**Figure 3:**
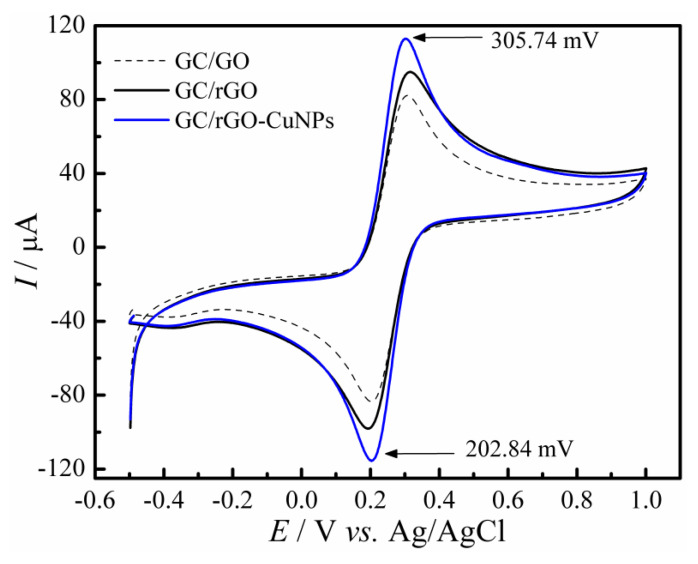
Cyclic Voltammograms recorded in 5.0 mmol L^−1^ [Fe(CN)_6_]^3−/4−^ containing 0.1 mol L^−1^ sulfuric acid solution for different modified electrodes.

To evaluate the GC/rGO-CuNPs sensor efficiency regarding its conductivity proprieties and to evidence the synergetic effect between rGO and CuNPs, a study with the ferricyanide/ferrocyanide redox probe was conducted. The following image, Figure 3, demonstrates the behavior of GC/rGO-CuNPs when comparing the CV scans to the GC/GO and GC/rGO sensors in a 5.0 mmol L^−1^ ferri/ferrocyanide solution, with 0.1 mol L^−1^ H_2_SO_4_ that was added to achieve voltammograms with better definition. With the resulting voltammogram, it is possible to see that, between the three tested electrodes, the GC/rGO-CuNPs showed a higher sensibility compared with GC/GO and GC/rGO; therefore, this modification is justified.

### 2.3. Fluoxetine Electrochemical Oxidation Processes

**Figure 4:**
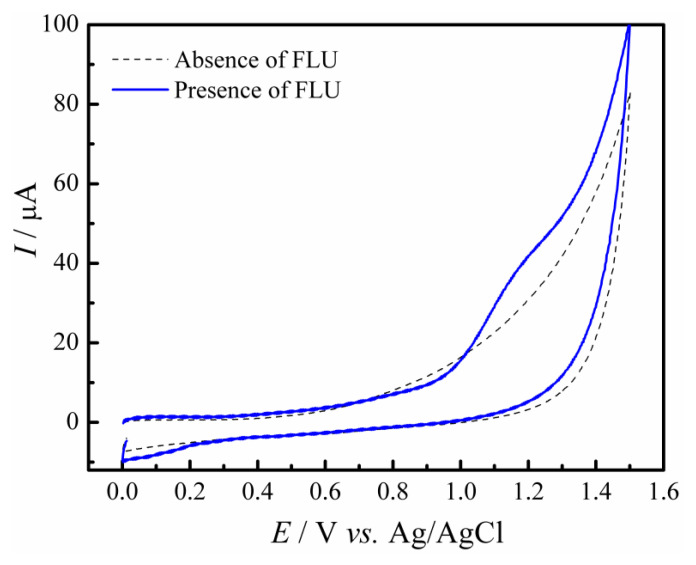
Cyclic voltammetry at the GC/rGO-CuNPs electrode in the absence (dashed line) and presence (solid blue line) of 100 µM of fluoxetine hydrochloride in a 0.1 mol L^−1^ PBS (pH 7) (*v* = 50 mV s^−1^).

Figure 4 shows the CV voltammograms obtained for the electrochemical oxidation of 100 µmol L^−1^ FLU in 0.1 mol L^−1^ PBS (pH 7). The dashed line represents the CV scan (*v* = 50 mV s^−1^) using the GC/rGO-CuNPs electrode in the supporting electrolyte. When FLU is present (solid line), an anodic peak at +1.15 V is observed. This irreversible oxidation processes were first reported by Lencastre et al. [37], and the results of this analysis are in accordance with their work.

Fluoxetine’s oxidation process was studied by Garrido et al. (2009) [38], in which they concluded that its oxidative process involves, mainly, its secondary amine group and an oxidation that occurs at the aromatic nucleus. This process originates an unstable cation-radical that “could undergo further subsequent reaction, such as dimerization” [38]. Moreover, it is worth mentioning that the presence of organic compounds based on an aromatic ring results in a highly complex oxidation process on electrodes based on carbon, since it involves “both the adsorption of the reagent/intermediate or oxidation products and the formation of passive, nonconductive layers of oligomer products of the oxidation process on their surface by electropolymerization” [39].

### 2.4. Evaluation of the Electrodes Modified with the Nanocomposites during the Fluoxetine Oxidation Process

**Figure 5:**
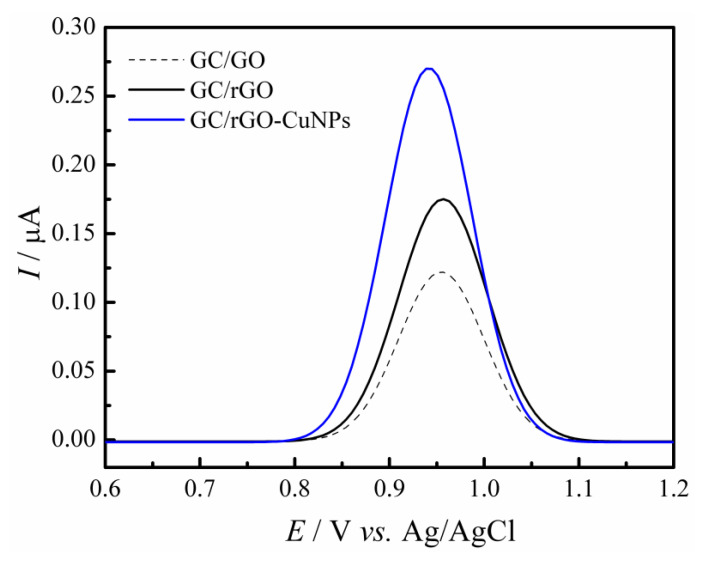
Comparison of different modified electrodes (GC/GO, GC/rGO and GC/rGO-CuNPs) at the oxidation process of 10 µmol L^−1^ of FLU by using DPV technique in 0.1 mol L^−1^ PBS (pH 7).

The DPV experiments were carried out in 0.1 mol L^−1^ PBS (pH 7) with 10 µmol L^−1^ of FLU. The working electrodes analyzed were GC/GO, GC/rGO, and GC/rGO-CuNPs. As shown in Figure 5, it is possible to observe that all sensors detected the oxidation peak of the drug; however, both GC/GO and GC/rGO do so to a lesser extent when compared to the GC/rGO-CuNPs. These data confirm a synergetic effect between rGO and the copper nanoparticles, which results in a higher anodic peak current for the FLU oxidation process, justifying the use of the GC/rGO-CuNPs for the identification of the drug and demonstrating their potential as a new material to be used in electrochemical analysis.

### 2.5. Analysis of the Optimization Parameters for the Detection of Fluoxetine in Response to the GC/rGO-CuNPs Electrode

**Figure 6:**
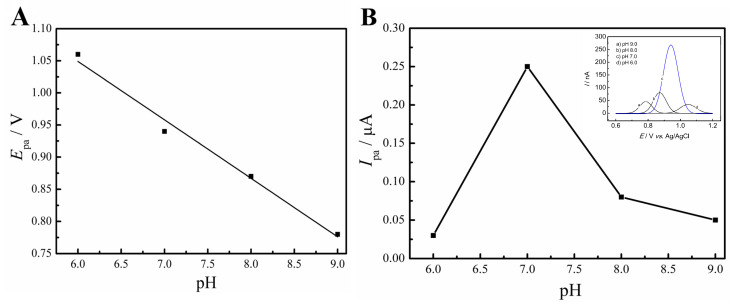
Influence of pH on the peak potential (*E_pa_*) (**A**) and anodic peak current (*I_pa_*) (**B**) for the oxidation of 10 µmol L^−1^ of FLU. Conditions: GC/rGO-CuNPs sensor, 0.1 mol L^−1^ PBS (pH = 6.0–9.0) (inset: voltammograms showing the voltammetric response for the different pHs).

In order to investigate the influence of pH variations in the oxidation behavior of FLU, DPV experiments were conducted using a 0.1 mol L^−1^ PBS with pH ranging between 6.0 and 9.0 (*v* = 10 mV s^−1^). A known amount of 10 µmol L^−1^ of the standard was employed. Figure 6 presents the obtained data, illustrating the changes in potential (*E*_pa_) and anodic peak current (*I*_pa_) across the pH range.

The *E_pa_* vs. pH plot shows a shift in the peak potential towards more negative values, resulting from the reduction in the hydrogen ion concentration (H^+^ ions) in the electrolyte. Such reduction is due to the deprotonation during the oxidation process, which is facilitated by more basic pH. In addition, a linear relationship between the peak potentials can be observed, with a coefficient of determination (R^2^) of 0.98.

The *I_pa_* vs. pH graph shows a sharp increase in the anodic peak current between the pH 6.0 and 7.0, the latter being the maximum value reached by the current, 0.25 µA, which is followed by a sharp drop in the peak current for pH 8.0 and 9.0. Therefore, the subsequent studies were performed with the 0.1 mol L^−1^ PBS solution pH 7.0.

It is known that the amount of metallic salt to be used in the synthesis influences the properties of the nanocomposite for the analysis of the analyte of interest. Our research group had carried out three studies before this one, which demonstrated that GC/rGO-CuNPs electrodes composed of 30% of the copper salt in the synthesis, concerning the amount of GO, present a higher peak current for the compounds: levofloxacin [40], glyphosate [41], and chloroquine phosphate [21]. Because the literature corroborates it, this study also adopted 30% GC/rGO-CuNPs electrodes.

### 2.6. Analytical Curve

**Figure 7:**
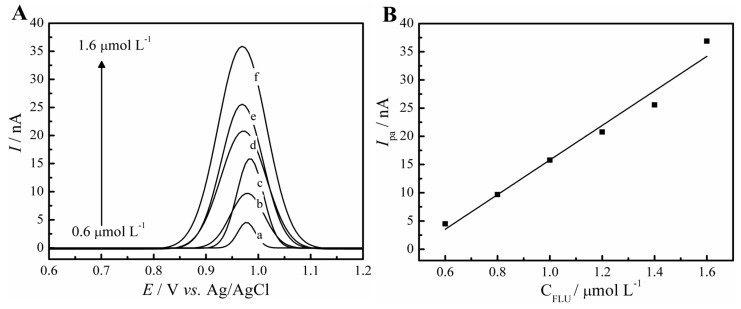
DPV experiments in 0.1 mol L^−1^ PBS (pH 7), (**A**) with the fluoxetine concentration being 0.6 μmol L^−1^ (a), 0.8 μmol L^−1^ (b), 1.0 μmol L^−1^ (c), 1.2 μmol L^−1^ (d), 1.4 μmol L^−1^ (e), and 1.6 μmol L^−1^ (f). (**B**) Linear dependence of the peaks current with fluoxetine concentrations.

The evaluation between the analytical response and the analyte concentration, and the verification of its linearity was performed with an analytical curve. The fluoxetine concentration varied from 0.6 to 1.6 μmol L^−1^, which was added to the 0.1 mol L^−1^ PBS pH 7.0 solution. The DPV technique was used once again, with a pulse amplitude of 25 mV, 10 mV s^−1^ of scan rate, and a potential window from +0.6 to +1.2 V. Figure 7 demonstrates the obtained anodic peak current values for the respective concentrations of fluoxetine and the linearity among the concentrations, whose equation can be described as follows:

*I*_pa_ (nA) = −14.86 (nA) + 30.66 (nA/µmol L^−1^) × C_FLU_ (µmol L^−1^)

The coefficient of determination (R^2^) for n = 6 was 0.97. Since this paper describes a new methodology to detect fluoxetine in phytotherapeutic drugs, the limit of detection (LOD) was also calculated, determining the lowest quantity of analyte that can be detected by the proposed method. This value is calculated based on the ratio of [3 × σ]/angular coefficient, with σ being obtained by the standard deviation of ten blank voltammograms. The value calculated was of 1.4 × 10^−7^ mol L^−1^.

While various techniques for detecting fluoxetine can be found in the literature, few experiments have been conducted using phytotherapeutic medicine as the medium. Table 1 provides valuable information on some of these studies. Indeed, the limit of detection (LOD) in the proposed article may not be the most sensitive compared to other techniques. However, it is important to note that most of these techniques did not specifically investigate weight loss herbal medicines. The choice of medium is crucial as it significantly influences the electrode’s performance and overall sensor efficacy. Compared to the LOD reported in [42], which also aimed to detect fluoxetine in herbal medicine samples, the technique presented in this study demonstrated slightly higher sensitivity. However, it is not as sensitive as the EGFET sensor described in [43], which detected fluoxetine in PBS. Additionally, copper, being less toxic and more cost-effective than noble metals, represents an alternative for developing low-cost devices suitable for large-scale applications [21].

The reproducibility of the proposed sensor for FLU analysis was measured from five experiments, in which each experiment consisted of ten sequential DPV voltammograms. These experiments were performed on different days. The relative standard deviation (RSD) was calculated as 2.6%. In addition, intra-assay precision tests were performed from ten DPV voltammograms of the same solution. The RSD was found to be 1.8%.

**Table 1:** Reported electrochemical sensors for FLU detection. Comparison of nanomaterials and LOD values.

Electrode	LOD (mol L^−1^)	Real Samples	Ref.
ZnO nanoparticles oriented MIP modified GCE	2.67 × 10^−12^	Tap water and spike serums	[26]
MIP ^1^ (itaconic acid monomer) modified GCE	3.33 × 10^−7^	Blood plasma	[27]
MIP (methacrylic acid monomer) modified CPE	2.8 × 10^−9^	Spiked plasma samples and fluoxetine capsules	[28]
BDD ^2^ electrode	1.07 × 10^−10^	Aqueous media	[39]
EGFET sensor ^3^	2.63 × 10^−12^	Phosphate-buffer saline (PBS)	[43]
PVC/PEDOT-C14-modified electrode ^4^	3.5 × 10^−8^	Tap and river water samples	[44]
BDD electrode	2.90 × 10^−7^	Thermogenic supplements, compounded drugs and weight loss herbal medicines	[42]
GC/rGO-CuNPs	1.4 × 10^−7^	Herbal medication	This work

^1^ Molecular imprinted polymer. ^2^ Boron-doped diamond. ^3^ Extended gate field-effect transistor. ^4^ Pencil lead modified with electrochemically deposited 3,4-ethylenedioxythiophene (PEDOT-C14) conductive polymer layer dipped coated with a plasticized poly(vinyl chloride) (PVC) membrane.

Studies can be found in the literature that conduct the detection of fluoxetine in weight loss herbal medicine using more traditional instrumental analysis techniques, such as chromatography and mass spectrometry. One example that can be cited is the work published by Kim et al. [45] in 2014, in which they monitored 29 weight loss compounds, including fluoxetine, in dietary supplements with LC-MS/MS (liquid chromatography tandem mass spectrometry). With this technique, the LOD found for FLU was 0.25 ng mL^−1^.

Both chromatography and mass spectrometry are more sensitive and selective techniques compared with electrochemical sensors; however, the latter counts with characteristics that should be taken into consideration when designing an experiment, such as easy automation, possibility of miniaturization, portability, and low cost [17]. Overall, even though electrochemical sensors are not as sensitive nor selective as chromatography and mass spectrometry, the elements obtained are enough for the decision-making process, making its use cost-effective.

### 2.7. Determination of Fluoxetine in Herbal Medication

**Figure 8:**
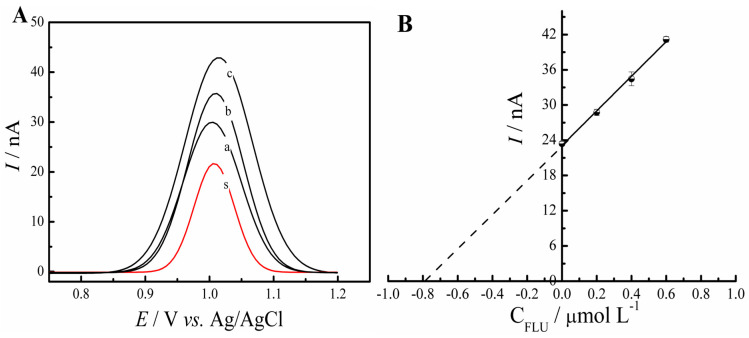
(**A**) DPV carried out in 0.1 mol L^−1^ PBS pH 7.0 with the sample(s), and the sample spiked with known concentrations of 0.2 µmol L^−1^ (curve a), 0.4 µmol L^−1^ (curve b), and 0.6 µmol L^−1^ (curve c) of the FLU standard. (**B**) Linear dependence of the peak current obtained for different FLU concentrations.

The samples in this step were prepared as described in Section 2.5, and the method employed in the analysis was the standard addition method. Therefore, three additions of FLU standard (0.2, 0.4, 0.6 µmol L^−1^) were enriched to 0.8 μmol L^−1^ of the analyte and then DPV experiments were run. Figure 8 demonstrates the resulting voltammogram and the linear relationship between *I*_pa_ values and FLU concentration. The results obtained of the triplicate (Table 2) show a mean of 0.797 ± 0.014 µmol L^−1^, with the recoveries ranging from 97.87% to 102.13%, indicating that the proposed electrode can be a low-cost and efficient alternative to determine fluoxetine in plant-derived medications.

### 2.8. Fluoxetine Oxidation Mechanism

**Figure 9:**
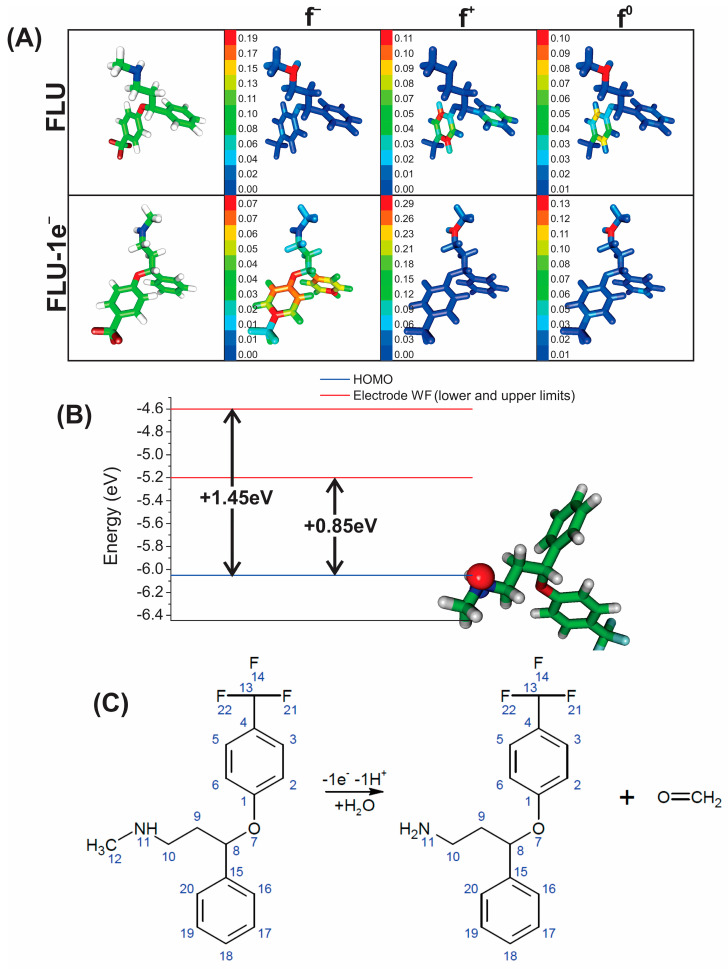
(**A**) Representation of fluoxetine 3D structure in its neutral form, FLU, and oxidized form, FLU–1e^−^, followed by the molecular structures colored by *f*^−^, *f*^+^, and *f*^0^ values from CAFIs (red and blue regions specify very high reactive and non-reactive sites, correspondingly). (**B**) Energy levels of HOMO from fluoxetine molecule (blue line) and work function range for rGO-CuNPs electrode (red lines). (**C**) Proposed pathway of the fluoxetine in the electrochemical experiments.

In Figure 9A, the atoms of the fluoxetine molecule (FLU) were colored according to a color scale defined by the CAFIs. The atoms colored in red on the *f^−^* index representation correspond to those most susceptible to losing an electron in an oxidation process. The results obtained from QM/DFT simulations showed that the fluoxetine atom most susceptible to losing an electron is the N11 (see numbering of atoms in Figure 9C). Figure 9B shows the HOMO spatial distribution of the fluoxetine molecule in its minimum energy state. In addition to the results obtained for the CAFIs, the HOMO indicated the N11 atom as the most susceptible to oxidation, revealing agreement between the two theoretical analyses (CAFIs and HOMO). The difference of energy (Δ*E*) between the fluoxetine HOMO (≈−6.05 eV) and the work function of the rGO-CuNPs electrode (which ranges from −5.20 eV to −4.60 eV) is consistent with the results obtained from electrochemical experiments (Figure 9B). Theoretical results show that Δ*E* can vary between +0.85 eV to +1.45 eV (depending on the electrode work function energy). Simultaneously, the oxidation peak of the fluoxetine molecule in the electrochemical experiments occurred at *E*_pa_ = +0.95 V.

The oxidation of fluoxetine makes the N11 atom very susceptible to nucleophilic or radical attack, as represented by the *f^+^* and *f^0^* indexes, respectively. This result is illustrated in Figure 9A for the fluoxetine molecule after losing one electron (FLU–1e^−^). This result agrees with a previous study that shows a possible dealkylation process of fluoxetine to produce a primary amine and formaldehyde. This process occurs precisely when the solution has a pH of 7 or higher due to hydroxyl ion (OH−) availability. With these results, it was possible to propose a fluoxetine oxidation pathway considering the formation of two products, as illustrated in Figure 9C. The formation of these products makes the oxidation process irreversible, which agrees with the electrochemical results and results previously reported in the literature [27,42].

## 3. Materials and Methods

### 3.1. Instrumentation

The electrochemical techniques, Cyclic Voltammetry (CV) and Differential Pulse Voltammetry (DPV), were carried out using a PGSTAT-128N Autolab electrochemical system (Ecochemie, Utrecht, The Netherlands) (NOVA 2.1.6 software).

A conventional three-electrode cell was assembled, consisting of a Ag/AgCl/KCl (3.0 mol L^−1^) as the reference electrode, a platinum (Pt) plate as the auxiliary electrode, and, at last, the working electrode (diameter: 3.0 mm), in which glassy carbon (GC) was used. The working electrode was modified either with graphene oxide (GO), reduced graphene oxide (GC/GO), or the newly synthesized reduced graphene oxide and copper nanoparticles (rGO-CuNPs) nanocomposite. All the experiments were carried out at room temperature (~25 °C) to avoid any temperature interference.

In order to investigate the voltammetric behavior of the modified electrodes, CV experiments were carried out in the potential range of 1.0 V to −0.5 V in a solution having 5.0 mmol L^−1^ ferricyanide/ferrocyanide redox probe [Fe(CN_6_)]^3−/4−^ and 0.1 mol L^−1^ H_2_SO_4_ (*v* = 50 mV s^−1^).

The presence of CuNPs onto rGO was characterized by CV experiments performed in 0.1 mol L^−1^ phosphate buffer solution (PBS) pH 7.0, with a scan rate of 50 mV s^−1^.

FLU analysis was carried out through DPV in 0.1 mol L^−1^ PBS (pH 7) (*v =* 10 mV s^−1^ and a pulse amplitude of 25 mV).

The morphological characterization of rGO and rGO-CuNPs was carried out in a Field Emission Gun—Scanning Electron Microscope, model Helios Nanolab 650 dual beam (Thermo Fisher Scientific, Waltham, MA, USA).

### 3.2. Reagents and Solutions

All solutions utilized in this experiment were prepared with purified water (PURELAB Q-ELGA-VEOLIA), while the reagents were of analytical grade and did not require further purification treatments.

The glassy carbon electrodes were polished with a solution of alumina 0.5 µm and, for the synthesis of the nanocomposites rGO and rGO-CuNPs, graphene oxide and copper chloride (CuCl_2_), both obtained from Sigma-Aldrich^®^, St. Louis, MO, USA; ethanol; the surfactant SDS (sodium dodecyl sulfate); and sodium borohydride (NaBH_4_) were used. Lastly, the fluoxetine used in the experiments was its pharmaceutical standard, fluoxetine hydrochloride.

The phytotherapeutic compound (herbal medication) consisting of 200 mg of Morosil^®^ (Moro red orange extract), 3 mg of capsiate, and 200 mg of Centella asiatica were obtained from a local manipulation pharmacy.

### 3.3. Synthesis of the Materials rGO and rGO-CuNPs

The rGO materials were obtained by following a previously described procedure by Barreto et al. [46]. A GO suspension (4.0 mg/mL, 5.0 mL) and sodium dodecyl sulfate (SDS) (8.0 mg) were added to ethanol (20 mL) and sonicated for 30 min. Sodium borohydride (NaBH_4_) (16.0 mg) was added. Then, the mixture was ultrasonicated for 30 min using an ultrasonic homogenizer. For rGO-CuNPs synthesis, CuCl_2_ was added at a concentration of 30% (m/m) relative to the mass of GO, diluted in ethanol, and added dropwise under constant stirring. The solution was sonicated for 1 h. Both composites were centrifuged at 3800 rpm for 5 min, and the supernatant was discarded. The composites were then deposited on Petri dishes and dried at 50 °C for 24 h. The dried materials were collected and used to prepare two suspensions: rGO and rGO-CuNPs dispersed in purified water, each at a concentration of 1.0 mg mL^−1^. To ensure homogeneity, both suspensions were sonicated for 10 min prior to electrode modification.

### 3.4. Working Electrode Cleaning and Modification Steps

The surface of the GC electrode was polished in a polishing cloth using 0.5 μm alumina suspension until a mirrored-like surface was achieved. Subsequently, the polished GC electrodes were sonicated in ethanol, then in water, for 5 min each.

After drying in room temperature, a 10 μL aliquot of the material of interest, at the concentration of 20 µg mL^−1^—GO, rGO, or rGO-CuNPs—was dripped on the electrode’s surface and dried at room temperature. Upon drying, the electrodes were ready to be used in the electrochemical procedures.

### 3.5. Preparation of the Plant-Derived Medication

**Figure 10:**
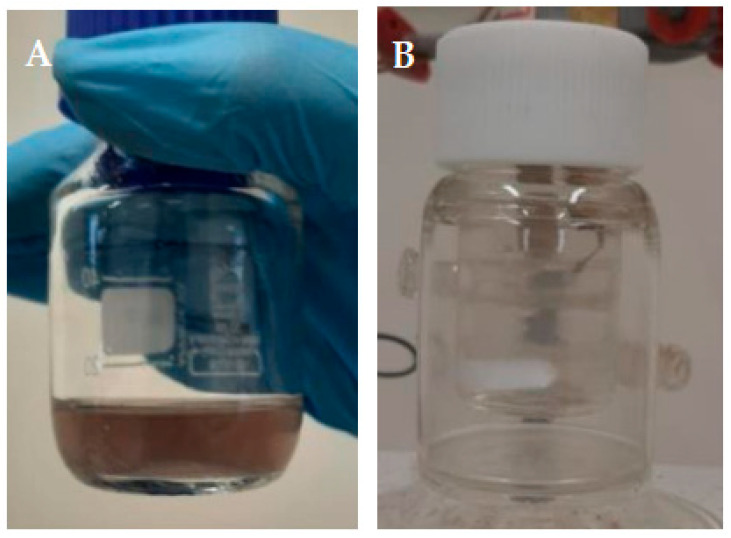
(**A**) First dilution. (**B**) Second dilution.

The herbal medication was decapsulated, and its content was quantified, resulting in a total of 0.5665 g. However, only 0.100 g of the sample was diluted in 20 mL of PBS pH 7.0. This decision was made because using the entire content of the capsule would have resulted in a highly concentrated solution, which could compromise the sensor’s efficiency. Even with the 0.100 g used, the solution remained highly concentrated, so 1 mL was taken and further diluted in 19 mL of PBS (pH 7) (Figure 10).

### 3.6. Theoretical Analysis

Theoretical analyses were carried out in order to compare the results obtained experimentally by the electrochemical measurements and the theoretical predictions for the oxidation potentials for FLU. Furthermore, these results were also used to describe the fluoxetine oxidation mechanism in electrochemical experiments. The conformational structure of the fluoxetine was obtained from the ChemSpider repository, with the following parameters: (i) molecular formula (C_17_H_18_F_3_NO); (ii) average mass: 309.326 Da; (iii) ChemSpider ID (3269). This molecule’s geometry and electronic structure were fully optimized via Density Functional Theory calculations using a quantum mechanical approach QM/DFT. Becke’s LYP (B3LYP) exchange-correlation functional and 6–31 G(p,d) basis set were employed. The Polarizable Continuum Model (PCM) was used to simulate the solvent’s presence at this stage. DFT calculations were performed with the Gaussian 09 computational package [47].

The evaluation of molecular oxidation susceptibility was accomplished with Condensed-to-Atoms Fukui Indexes (CAFIs): (i) *f_k_*^+^ = *q_k_* (*N* + 1) − *q_k_* (*N*) for nucleophilic attack on atom *k*; (ii) *f_k_*^−^ = *q_k_* (*N*) − *q_k_* (*N* − 1) for electrophilic attack on atom *k*; and (iii) *f_k_*^0^ = ^1^/_2_[*q_k_* (*N* + 1) − *q_k_* (*N* − 1)] for radical attack on atom *k*. Furthermore, *q_k_* (*N* + 1), *q_k_* (*N*), and *q_k_* (*N* − 1) represent the electronic Hirshfeld population on the *k-th* atom of anionic, cationic, and neutral species, respectively, of the studied compound [48,49,50]. CAFIs have been successfully used to identify oxidation, reduction, and charge exchange mechanisms in chemical reactions [51,52,53]. In addition, the location and spatial distribution of frontier molecular orbitals, specifically the highest occupied molecular orbital (HOMO), was illustrated using the Gabedit computational package [54].

## 4. Conclusions

The study successfully synthetized and characterized rGO-CuNPs, and developed an electrochemical sensor based on this material. The sensor intended to detect fluoxetine, an antidepressant, in plant-derived medication samples and did it effectively, with a superior response at a pH of 7.0.

The analytical curve demonstrated the sensor’s capability to detect fluoxetine at concentrations as low as 0.6 µmol L^−1^. However, when the standard addition method was employed in herbal medicine samples, the sensor detected the fluoxetine at concentrations starting from 0.8 µmol L^−1^. The slight variation can be attributed to the complex matrix of the medication, but, even considering how the matrix could have interfered with the analysis, this experiment had positive results: the initial concentration detected can still be considered low, and the standard addition was conducted in triplicate, demonstrating that the results can be replicated. Additionally, the sensor maintained its stability throughout the entire experiment, being a reliable method for analysis.

Furthermore, theoretical findings elucidated an irreversible oxidation process involving the dealkylation of fluoxetine, producing a primary amine and formaldehyde.

Further investigations could explore more refined sample preparation techniques to assess their influence on the sensor’s sensitivity towards fluoxetine. Additionally, studies focused on optimizing the electrode itself would be valuable. It is important to note that the effectiveness of this method may vary depending on the diverse formulations of the countless phytotherapeutic drugs available in the market, which may or may not interfere with the proposed methodology.

## Figures and Tables

**Table 2:** Results of the determination of FLU in plant-derived medication using the GC/rGO-CuNPs electrodes with the DPV method.

Repetition	Fluoxetine (μmol L^−1^) ^a^	Relative Errors ^b^
1	0.817	2.13
2	0.792	−1.00
3	0.783	−2.13
Mean ± SD	0.800 ± 0.014	

^a^ Added value for FLU: 0.8 μmol L^−1^. ^b^ DPV vs. added (DPV—added/added) × 100%.

## Data Availability

Data are available from the authors upon reasonable request.
